# Identification of *KDM4C* as a gene conferring drug resistance in multiple myeloma

**DOI:** 10.1515/biol-2022-0848

**Published:** 2024-04-12

**Authors:** Na Zhang, Ruilong Lan, Yingyu Chen, Jianda Hu

**Affiliations:** Fujian Institute of Hematology, Fujian Provincial Key Laboratory on Hematology, Fujian Medical University Union Hospital, Fuzhou, 350001, China; Fujian Medical University Center of Translational Hematology, Fuzhou, 350001, China; Central Lab, The First Affiliated Hospital of Fujian Medical University, Fuzhou, 350005, China

**Keywords:** KDM4C, drug resistance, multiple myeloma, bortezomib, proteasome inhibitors, hematologic cancer

## Abstract

Bortezomib (BTZ), a proteasome inhibitor, is a promising therapeutic option for multiple myeloma (MM) patients. However, drug resistance often occurs, leading to disease relapse and poor prognosis. In this study, we aimed to identify novel genes associated with drug resistance and investigate their roles in BTZ resistance. Through the screening of 26 genes frequently associated with chemosensitivity or drug resistance, we discovered that KDM4C, a histone demethylase, exhibited increased expression in BTZ-resistant MM cells compared to their sensitive counterparts. Overexpression of *KDM4C* enhanced the tolerance of a MM cell line to the drug, whereas the knockdown of *KDM4C*, using shRNA, increased the sensitivity of resistant cells to BTZ treatment. This suggests that KDM4C plays a pivotal role in conferring BTZ resistance. Our study offers fresh insights into BTZ resistance in MM and highlights *KDM4C* as a potential target for overcoming drug resistance.

## 1 Introduction

Multiple myeloma (MM) is a malignant neoplasm of plasma cells, which accounts for approximately 1% of all cancers and 10% of hematological malignancies [1]. Bortezomib (BTZ), a proteasome inhibitor, has revolutionized the treatment of MM by inducing apoptosis and inhibiting cell proliferation [2]. Despite its effectiveness, resistance to BTZ frequently develops, resulting in disease relapse and a poor prognosis [3,4]. Consequently, understanding the mechanisms underlying BTZ resistance is crucial for enhancing therapeutic outcomes.

Several genes have been identified as contributing to the development of BTZ resistance in MM, such as *MDR1*, *BCL-2*, and *NF-κB* [5]. However, identifying novel genes that confer resistance to BTZ is essential for developing effective therapeutic strategies. In this study, we examined a group of 26 genes frequently associated with chemosensitivity or drug resistance, with the goal of identifying novel genes involved in BTZ resistance (Table 1).

**Table 1:** List of genes analyzed for expression in cell lines. A set of 26 genes, frequently associated with chemosensitivity or drug resistance, was screened to identify novel genes in BTZ resistance

No.	Gene	Reference*
1	KDM4C	doi: 10.1038/s41419-020-03380-2
2	MDR1	doi: 10.1016/j.ejmech.2019.05.027
3	Bax	doi: 10.15252/embr.201745235
4	Bcl-2	doi: 10.1016/j.bbcan.2021.188569
5	LC3	doi: 10.1080/01913123.2017.1419328
6	Beclin-1	doi: 10.1002/1878-0261.12641
7	cyclinD	doi: 10.1080/14737140.2020.1834385
8	CDK4	doi: 10.1158/1078-0432.CCR-21-2947
9	JAK2	doi: 10.3389/pore.2022.1610273
10	STAT3	doi: 10.1016/j.biopha.2019.109135
11	JAG1	doi: 10.1002/jcb.26469
12	β-catenin	doi: 10.1158/2159-8290.CD-19-0074
13	TMEM132A	doi: 10.3389/fcell.2020.599890
14	B23	doi: 10.1016/j.imbio.2014.10.015
15	GLUT1	doi: 10.1007/s00210-020-01893-3
16	HIF-1α	doi: 10.3390/cancers14246054
17	HK2	doi: 10.1158/0008-5472.CAN-21-1820
18	LDHA	doi: 10.1158/0008-5472.CAN-14-3400
19	LDHB	doi: 10.3390/genes12050777
20	PDHA1	doi: 10.3389/fcell.2021.738916
21	CKMT1	doi: 10.1080/09553002.2019.1554919
22	GRB7	doi: 10.1186/s12943-021-01476-7
23	PASK	doi: 10.18632/aging.102745
24	NF-κB	doi: 10.1530/ERC-19-0087
25	p53	doi: 10.1016/j.drup.2019.100671
26	c-Myc	doi: 10.1016/j.canlet.2018.11.030

*Selected references linking the gene to drug resistance or chemosensitivity.

**Figure 1:**
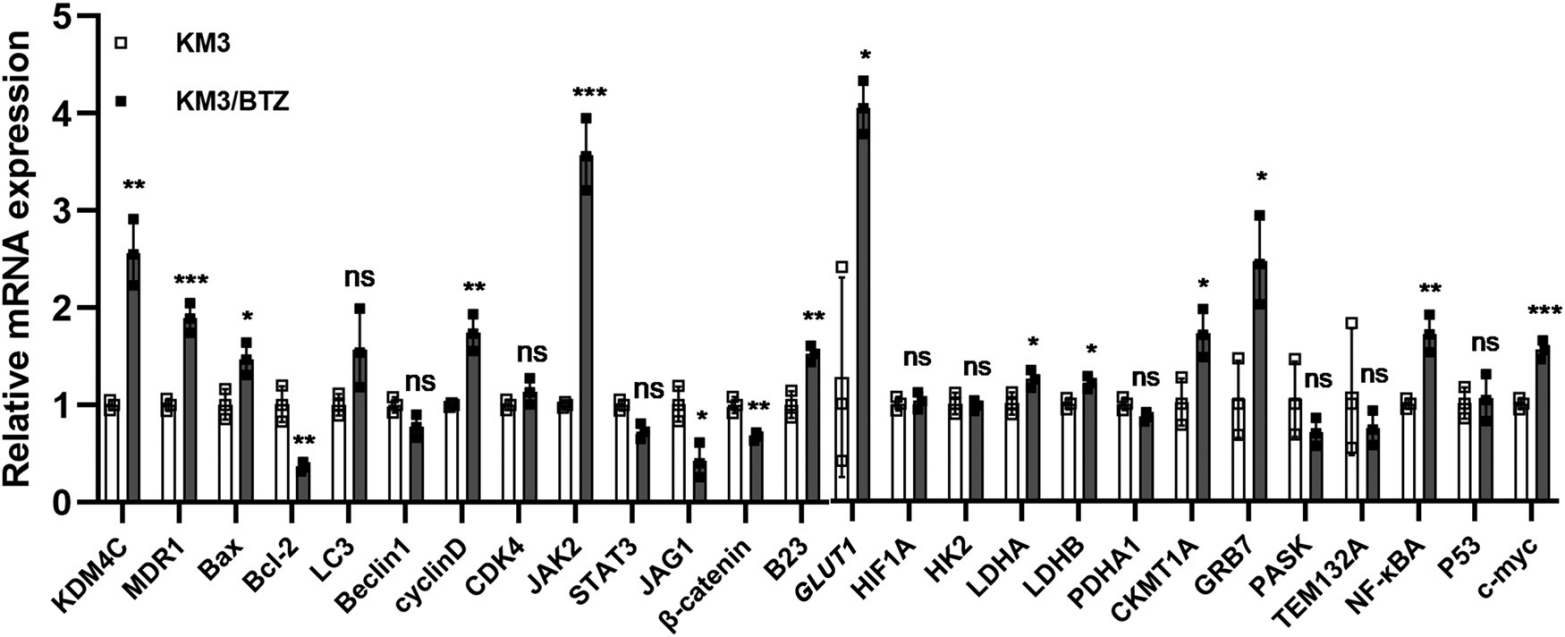
Relative gene expression in cell lines. Compared to KM3, *KDM4C, MDR1, Bax, cyclinD, JAK2, B23, GLUT1, LDHA, LDHB, CKMT1A, GRB7, NF-κB*, and *c-Myc* mRNA levels were upregulated and *Bcl-2, JAG1*, and *β-catenin* mRNA levels were decreased in the KM3/BTZ.

Our findings revealed an upregulation of KDM4C in BTZ-resistant MM cells (Figure 1). Additionally, we found that reducing its expression made the cells more sensitive to BTZ treatment. These results suggest that KDM4C, a histone demethylase, may be a promising therapeutic target for combating BTZ resistance in MM.

This study offers new understanding into the molecular mechanisms behind BTZ resistance in MM and highlights *KDM4C* as a potential gene related to drug resistance. Further insight into KDM4C’s role in BTZ resistance could guide the development of innovative therapeutic strategies, with the aim of enhancing clinical outcomes for patients with MM.

## 2 Materials and methods

### 2.1 Cell culture and transfection

The human MM cell lines KM3 and KM3/BTZ were provided by Qilu Hospital of Shandong University. KM3 cells were cultured in RPMI-1640 medium, supplemented with 10% fetal bovine serum. The cells were maintained at a temperature of 37°C in a 5% CO_2_ humidified atmosphere. KM3/BTZ cells were established as follows [6]: parent KM3 cells in logarithmic growth phase were subjected to gradient induction with BTZ at concentrations ranging from 5 to 160 ng/mL, then sustained in 5 ng/mL. After 10 months of this treatment, the BTZ-resistant cell line, KM3/BTZ, was successfully established. Lentivirus for overexpression and knockdown of KDM4C (NM_015061) were obtained from Shanghai Genechem Co., Ltd. Cells were plated at a density of 5 × 10^4^ cells/well in 500 μL of complete medium in 24-well plates. The lentivirus was added to the cells at a multiplicity of infection ratio of 1:100. After 12 h, the medium was replaced. The shRNA sequences used for targeting KDM4C were as follows: scramble sequence: TTCTCCGAACGTGTCACGT; sh1: AGGAGTTCCGGGAGTTCAACA; sh2: GCAAGTACTGGAAGAACTTAA.

### 2.2 Real-time quantitative PCR

Total RNA was isolated from cell lysates utilizing the RNA-easyTM isolation reagent (Nanjing Vazyme BioTech CO., Ltd, R711-01). Complementary DNA was then synthesized from the extracted RNA using the HiscriptII Q RT Supermix for qPCR (Vazyme, R223-01). The mRNA expressions of target genes were quantified using a SYBR green PCR master mix (abm, G891-1). The PCR amplification was carried out on a QuantaStudio5 real‐time PCR system (Applied biosystems by Thermo Fisher Scientific). Each PCR experiment was conducted in triplicate. The relative mRNA levels of target genes were calculated using the 2^−ΔΔCt^ method, with *β-ACTIN* as the internal control.

Primer sequences were as follows:

*KDM4C*-F: 5′-CATGGAGTCTAAAGGAGCCCA-3′;

*KDM4C*-R: 5′-TGTACTGAGTGAACAGTCCTGA-3′;

*MDR1*-F: 5′-CCCATCATTGCAATAGCAGG-3′;

*MDR1*-R: 5′-GTTCAAACTTCTGCTCCTGA-3′;

*β-ACTIN*-F: 5′-CTGGAACGGTGAAGGTGACA-3′;

*β-ACTIN*-R: 5′-AAGGGACTTCCTGTAACAACGCA-3′.

### 2.3 Western blotting

Cells were lysed using pierce IP lysis buffer (Thermo scientific, REF: 89900), supplemented with phosphatase inhibitors (MCE, HY-K0021), protease inhibitor (MCE, HY-K0010), and phenylmethanesulfonyl fluoride (MCE, HY-B0296/CS-2617). Proteins were separated by SDS-PAGE gel based on their molecular weight. The gel was then transferred onto a polyvinylidene fluoride (PVDF) membrane using a semi-dry method. After the transfer, the PVDF membrane was blocked with 5% BSA in tris buffered saline with tween 20 (TBST). The membrane was then incubated with primary antibodies, including anti-MDR1 (CST, #13978S), KDM4C (ABclonal, A8485), β-TUBULIN (CST, #81253SF), β-ACTIN (CST, #4970S) diluted with 1% BSA at 4°C overnight. After incubation, the membrane was washed three times with TBST and then incubated with HRP-conjugated secondary antibody for 1 h at room temperature. Enhanced chemiluminescence substrates were added to the membrane and images were captured and developed by a FluoChem E system (Proteinsimple). The protein levels were normalized by β-TUBULIN or β-ACTIN. All experiments were performed three times.

### 2.4 IC_50_ assay

The drug susceptibility of the cells was evaluated using the 3-(4,5-dimethylthiazol-2-yl)-2,5-diphenyltetrazolium bromide (MTT, Solarbio, 298-93-1) assay. MM cells (5 × 10^3^ cells/well) were seeded into 96-well plates (Nest), and were incubated with different concentrations of BTZ at 37°C in a humidified incubator containing 5% CO_2_ for 48 h. The BTZ concentrations for KM3 and KM3/BTZ were 0, 5, 10, 20, 50, 100, 200, and 400 ng/mL. BTZ concentrations for empty vector (EV) and overexpression (OE) cells were 0, 5, 10, 20, 40, and 80 ng/mL. BTZ concentrations for scramble, sh1, and sh2 cells were 0, 60,120, 240, 480, and 960 ng/mL. After the incubation period, 20 µL of MTT (5 mg/mL in phosphate-buffered saline) was added to each well and incubated for 4 h. The supernatant was then carefully removed, and 100 µL of dimethyl sulfoxide was added to dissolve the formazan crystals that had formed. The absorbance of each well was then measured at 490 nm using a CLARIOstar^Plus^ spectrophotometer. Cell survival and inhibitory rates were derived as (optical density [OD]_treated_/OD_control_)  ×  100% and (1 − OD_treated_/OD_control_)  × 100%, respectively. IC_50_ was calculated by GraphPad Prism 9 software. All experiments were carried out in triplicate.

### 2.5 Intracellular ADM detection by flow cytometry

Intracellular ADM (Adriamycin) accumulation was evaluated by flow cytometry (BD FACS Celesta). 1.0 × 10^6^ cells were cultured with or without 1 μg/mL ADM for 9 h. The ADM fluorescence intensity was analyzed by FlowJo Software Version 10. Each experiment was repeated three times.

### 2.6 Statistical analysis

Data are presented as the mean ± standard deviation. Differences between groups were evaluated with *T*-test by GraphPad Prism 9 software. *p* < 0.05 was considered to be a statistically significant difference (*p < 0.05, **p < 0.01, *** *p* < 0.001).


**Ethical approval:** The conducted research is not related to either human or animals use.

## 3 Results

### 3.1 KDM4C was upregulated in the BTZ-resistant MM cell line KM3/BTZ

**Figure 2:**
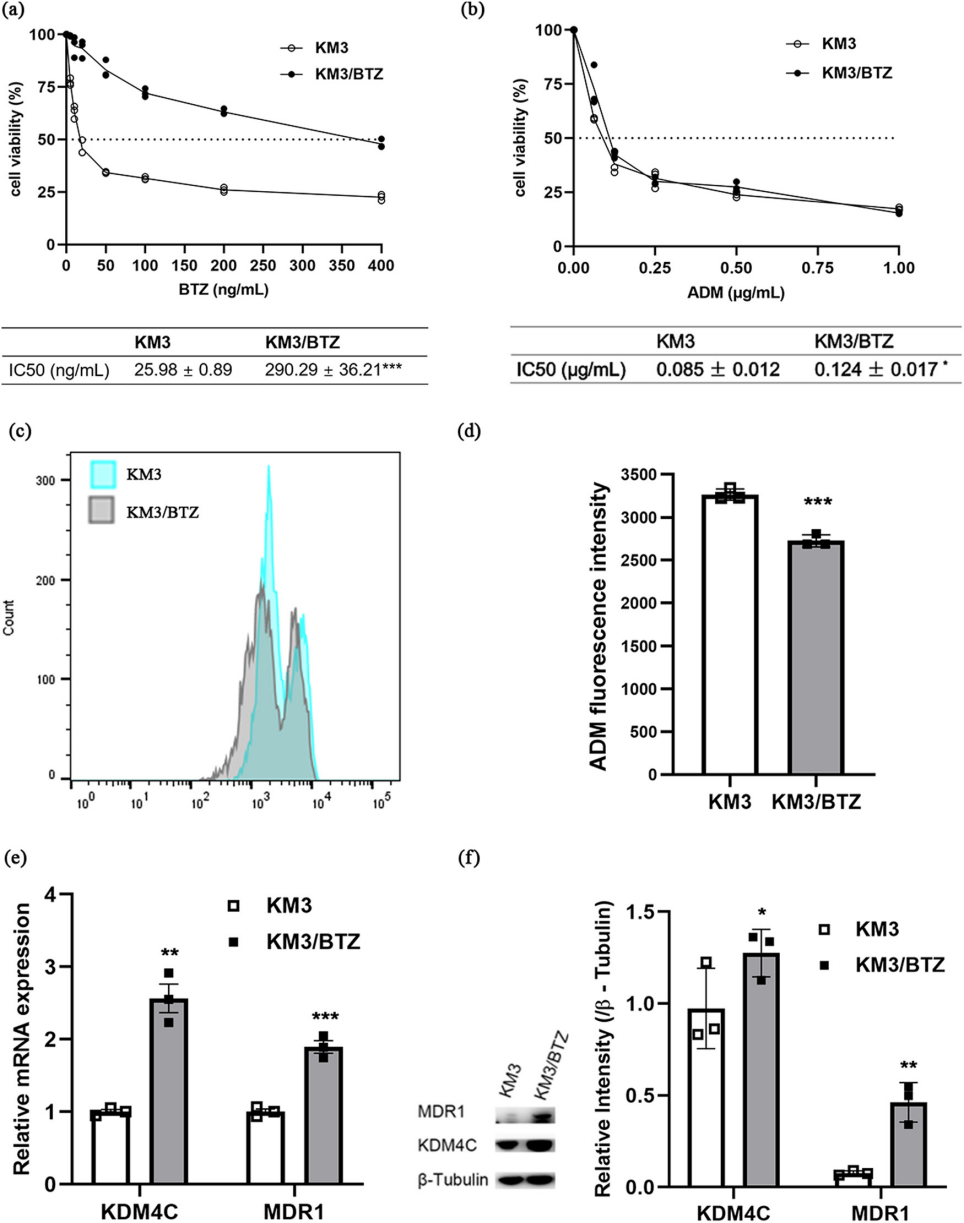
KDM4C was upregulated in KM3/BTZ cells. (a) Survival curves and BTZ IC_50_ values of KM3 and KM3/BTZ. (b) Survival curves and IC_50_ values of KM3 and KM3/BTZ for ADM. (c) Representative plots showing intracellular ADM accumulation in KM3 and KM3/BTZ. (d) ADM fluorescence intensity in KM3 and KM3/BTZ cells were determined by flow cytometry. (e) mRNA levels of KDM4C and MDR1 in KM3 and KM3/BTZ. (f) Protein levels of KDM4C and MDR1 in KM3 and KM3/BTZ.

KM3/BTZ is a BTZ-resistant human MM cell line, derived from the MM cell line KM3. The BTZ IC_50_ was found to be 290.29 ng/mL for KM3/BTZ. This is 11.17 times higher than that of the original parent cell line, KM3 (*p* < 0.001, Figure 2a). Furthermore, KM3/BTZ shows cross-resistance to ADM (Figure 2b). ADM accumulation in drug-resistant KM3/BTZ cells was significantly lower than in the KM3 cells (Figure 2c and d). MDR1, also known as P-glycoprotein /ABCB1, is a prominent member of ABCB (encoded by *ABCB1* gene) subfamily and is a membrane-embedded drug transporter. MDR1, commonly overexpressed in cancers, is considered as a primary factor in the induction of multidrug resistance. It is the leading causes of various mechanisms of multidrug resistance [7]. As we anticipated, the levels of MDR1 mRNA and protein were significantly elevated in KM3/BTZ (Figure 2e and f). Meanwhile, we identified that the histone demethylase KDM4C was significantly elevated in KM3/BTZ (Figure 2e and f). KDM4C (also known as JMJD2C/GASC1) consists of a conserved JmjC catalytic domain and has been shown to demethylate lysine 9 of histone H3 (H3K9me2 and H3K9me3) and lysine 36 of histone H3 (H3K36me2 and H3K36me3). KDM4C could activate target genes by binding to their promoters and removing trimethyl (me3) and dimethyl (me2) groups [8,9]. *KDM4C* is amplified or overexpressed in many human cancers and is closely associated with the degree of malignancy of the tumors [10–19]. Given the elevated levels of KDM4C in KM3/BTZ, we questioned whether KDM4C plays a role in BTZ resistance in MM cells.

### 3.2 *KDM4C* overexpression enhanced BTZ resistance in KM3

**Figure 3:**
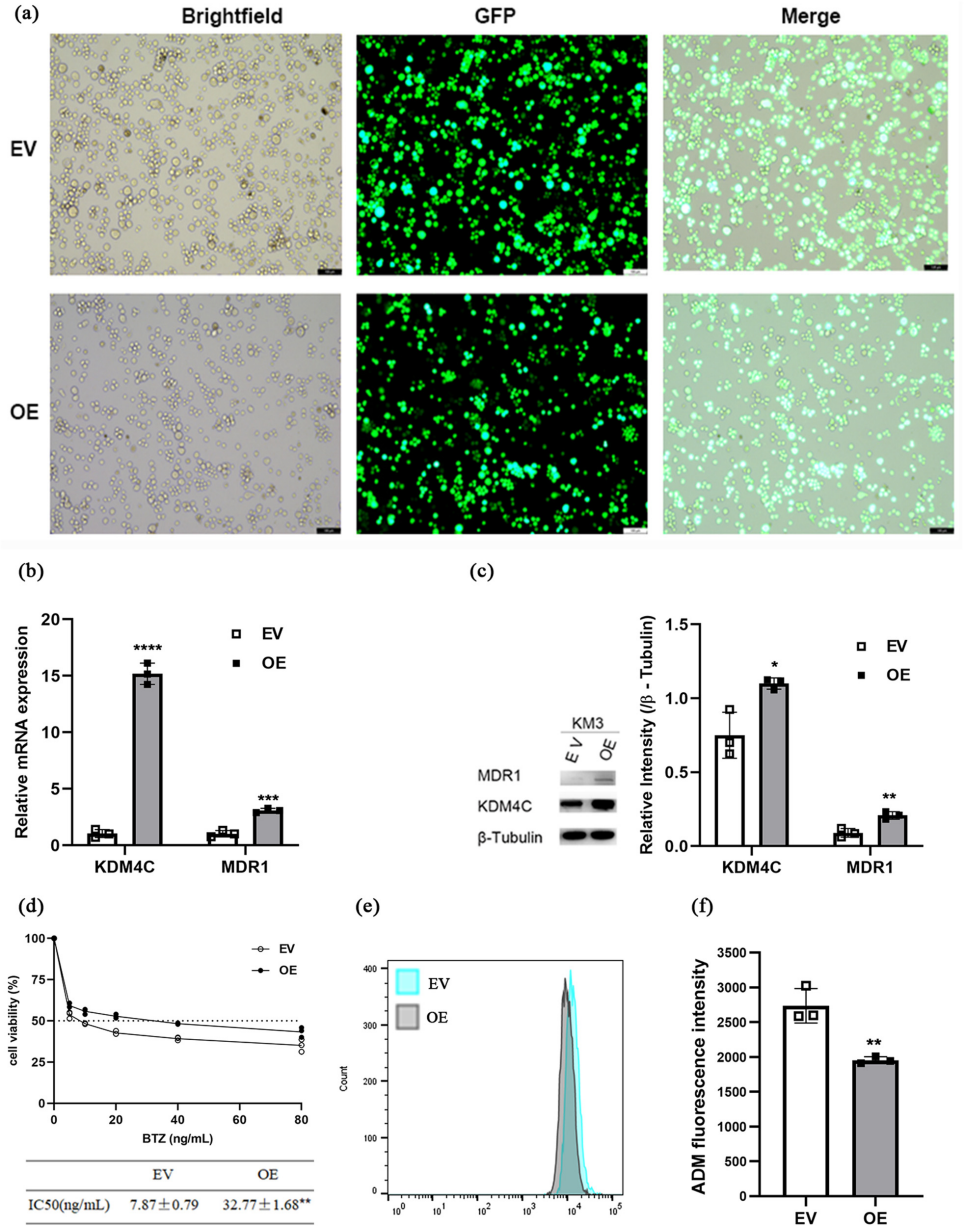
*KDM4C* overexpression in KM3 promoted BTZ resistance. (a) Lentivirus transfection efficiency in KM3 cells, assessed by fluorescence microscopy (magnification, ×100; scale bar  =  100 μm). (b and c) KDM4C overexpression was associated with increased MDR1 mRNA and protein expression. (d) Survival curves and BTZ IC_50_ of OE and EV. (e) Representative flow cytometry plots showing ADM accumulation in EV and OE group. (f) ADM fluorescence intensity was decreased in OE group.

To explore whether KDM4C is connected to BTZ resistance in MM cells, KM3 cells were transfected with either an EV or a *KDM4C* OE lentivirus (Figure 3a). RT-PCR and western blot assays were used to confirm that the KDM4C level in the OE group was significantly higher than that in the EV group (Figure 3b and c). As anticipated, we observed a significantly higher expression of MDR1 in the OE group (Figure 3b and c). Similarly, the MTT assay showed that the IC_50_ of BTZ in the OE group was significantly higher than that in the EV group (32.77 ng/mL vs 7.87 ng/mL, *p* < 0.01, Figure 3d). The intracellular ADM fluorescence intensity was found to be decreased in the OE group (Figure 3e and f). These findings suggest that KDM4C may be associated with BTZ resistance in MM cells.

### 3.3 *KDM4C* knockdown reduced the resistance to BTZ in KM3/BTZ

**Figure 4:**
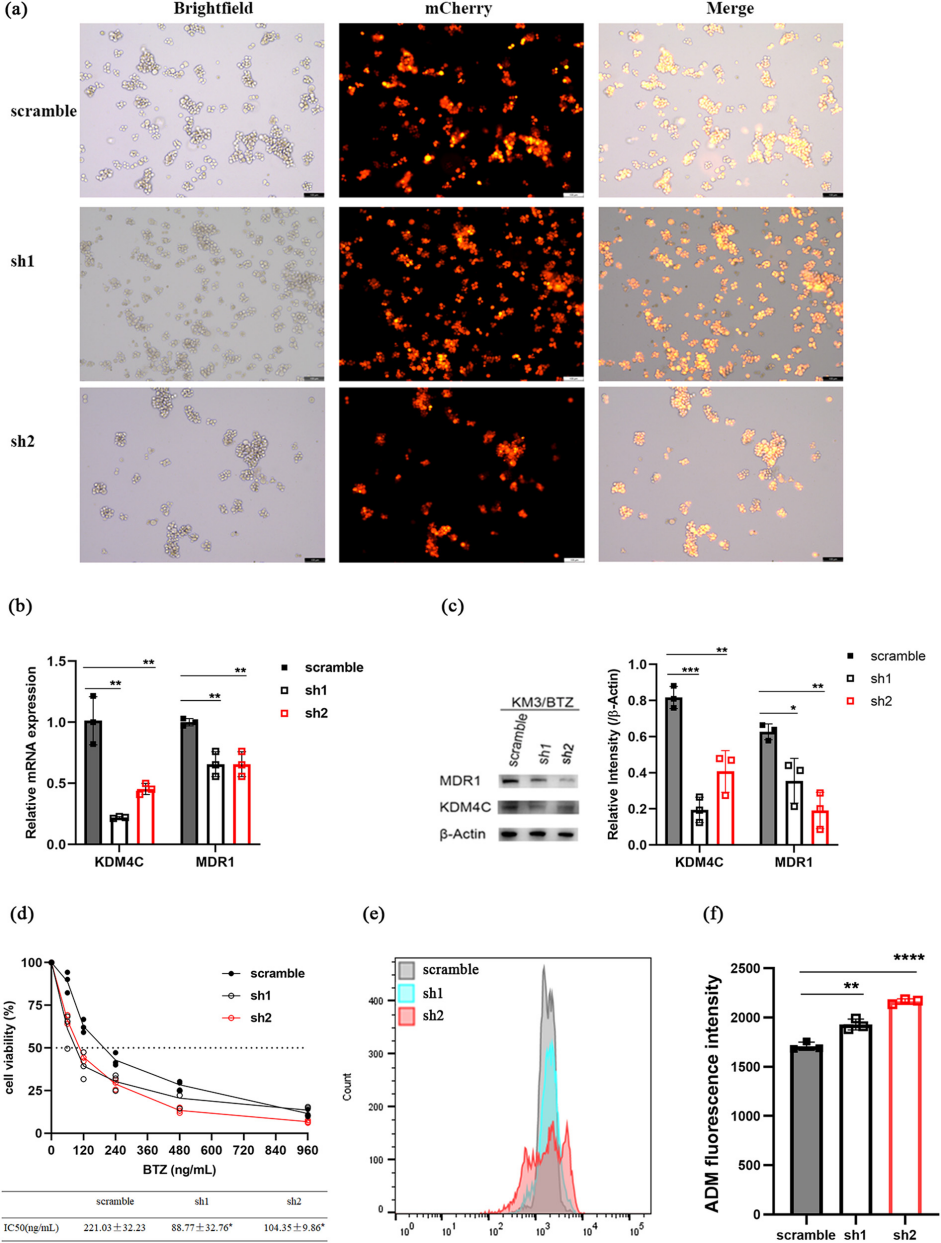
*KDM4C* knockdown attenuated BTZ resistance in KM3/BTZ. (a) Verification of lentivirus transfection efficiency, with mCherry expression observed in >90% of cells using fluorescence microscopy (magnification, ×100; scale bar  =  100 μm). (b and c) mRNA and protein levels of MDR1 after KDM4C knockdown in KM3/BTZ. (d) Survival curves and BTZ IC_50_ of scramble, sh1 and sh2 groups. (e) Representative flow cytometry plots showing ADM accumulation in scramble, sh1 and sh2 groups. (f) Intracellular ADM accumulation was increased in sh1 and sh2.

To further investigate the role of KDM4C in BTZ resistance, two different KDM4C knockdown cell lines were developed using two different lentivirus-mediated shRNAs in KM3/BTZ. A scramble group was used as a control (Figure 4a). The knockdown of KDM4C in KM3/BTZ, as predicted, led to a decrease in MDR1 expression (Figure 4b and c), The knockdown also decreased the IC_50_ of BTZ (from 221.03 ng/mL to either 88.77 or 104.35 ng/mL, both *p* values <0.05, Figure 4d) and increased ADM accumulation (Figure 4e and f). The collected data strongly suggest that KDM4C plays a role in promoting resistance to BTZ in MM cells.

The BTZ-resistant MM cell line KM3/BTZ showed an upregulation of KDM4C. Furthermore, overexpression of *KDM4C* resulted in an increase in the BTZ IC_50_ for the wild type MM cell line KM3. Conversely, knockdown of *KDM4C* led to a decrease in BTZ IC_50_ in the BTZ-resistant MM cell line KM3/BTZ. Consequently, we have established a connection between KDM4C and BTZ resistance in MM cells.

## 4 Discussion

In this study, we discovered a previously unidentified role of KDM4C in mediating drug resistance in a MM cell line. Our study revealed that KDM4C expression is elevated in BTZ-resistant MM cells and that depleting its levels increases the susceptibility of these cells to BTZ therapy. These significant findings strongly propose a regulatory role for KDM4C in imparting BTZ resistance in MM.

The aforementioned findings necessitated a deeper exploration into the potential mechanisms underlying the connection between KDM4C and MDR1 in MM cells. As a histone demethylase, KDM4C could potentially engage in a mechanism: it is plausible that KDM4C may bind to the promoter region of MDR1, thereby stimulating its transcription and leading to enhanced drug resistance in MM. However, no pertinent literature reports have been identified to support this hypothesis, necessitating further experimental verification.

KDM4C may promote BTZ resistance in MM cell lines by affecting the expression of MDR1. Apart from this, KDM4C is known to govern various cellular pathways. It plays a pivotal role in the DNA damage response [20], moderating cell cycle progression [21], directing differentiation [22], and maintaining the characteristics of tumor stem cells [23,24]. Hence, KDM4C could potentially influence BTZ resistance by impacting some of these pathways. The role of KDM4C in drug resistance may depend on the specific disease context and warrants further research. KDM4C plays a crucial role in various cancers, including lung cancer, hepatocellular carcinoma, ovarian cancer, and acute myeloid leukemia (AML). Overexpression of KDM4C has been shown to promote radioresistance in lung cancer and hepatocellular carcinoma [25,26]. Additionally, KDM4C is involved in maintaining the stem cell properties of cancer cells in ovarian cancer and AML [23,24,27]. Lowering the expression of KDM4C has been found to induce cellular senescence in JAK2-mutated neoplasms and gastric cancer with TP53 mutations [28,29]. It is important to note that KDM4C may also play a role in resistance to other medications. For example, KDM4C has been found to mediate resistance to cytarabine in AML [30]. However, further research is needed to fully understand the role of KDM4C in cancers and to develop effective KDM4C-targeted therapies.

Targeting KDM4C could potentially be a therapeutic strategy for overcoming BTZ resistance in MM. Several molecules that inhibit KDM4C activity, such as JIB-04 [31] and SD70 [32], have been created and demonstrated anti-tumor effects in preclinical studies. As such, these molecules could potentially enhance the effectiveness of BTZ treatment in MM patients who are resistant to BTZ.

In conclusion, this study has shed new light on the molecular mechanisms underlying BTZ resistance in MM by highlighting the role of KDM4C as a key gene involved in conferring drug resistance. These findings have significant implications for the development of innovative therapeutic approaches to overcome BTZ resistance. Further investigations are warranted to fully elucidate the precise mechanisms through which KDM4C mediates BTZ resistance. Moreover, future studies should prioritize the evaluation of the therapeutic potential of KDM4C inhibitors, such as JIB-04 and SD70, in the treatment of MM patients.
